# The Role of Sleep and Sleep Disorders in the Development, Diagnosis, and Management of Neurocognitive Disorders

**DOI:** 10.3389/fneur.2015.00224

**Published:** 2015-10-23

**Authors:** Michelle A. Miller

**Affiliations:** ^1^Warwick Medical School, University of Warwick, Coventry, UK

**Keywords:** sleep, sleep disorders, cognition, dementia, neurocognitive disorders

## Abstract

It is becoming increasingly apparent that sleep plays an important role in the maintenance, disease prevention, repair, and restoration of both mind and body. The sleep and wake cycles are controlled by the pacemaker activity of the superchiasmic nucleus in the hypothalamus but can be disrupted by diseases of the nervous system causing disordered sleep. A lack of sleep has been associated with an increase in all-cause mortality. Likewise, sleep disturbances and sleep disorders may disrupt neuronal pathways and have an impact on neurological diseases. Sleep deprivation studies in normal subjects demonstrate that a lack of sleep can cause attention and working memory impairment. Moreover, untreated sleep disturbances and sleep disorders such as obstructive sleep apnoe (OSA) can also lead to cognitive impairment. Poor sleep and sleep disorders may present a significant risk factor for the development of dementia. In this review, the underlying mechanisms and the role of sleep and sleep disorders in the development of neurocognitive disorders [dementia and mild cognitive impairment (MCI)] and how the presence of sleep disorders could direct the process of diagnosis and management of neurocognitive disorders will be discussed.

## Background

The worldwide prevalence of neurocognitive disorders [dementia and mild cognitive impairment (MCI)] is increasing. Dementia occurs when the brain is damaged and is characterized by memory loss, alongside thinking, problem-solving, or language difficulties.

Alzheimer’s disease (AD) is a neurodegenerative disorder in which there is a progressive decline in cognitive function. It is the most common cause of dementia. In 2010, the worldwide prevalence of AD was estimated to be 35.6 million (1). While the worldwide prevalence for those aged 60 or above is estimated between 5 and 7%, a higher prevalence in Latin America (8.5%) and a distinctively lower prevalence in the four sub-Saharan African regions (2–4%) are reported. The number of cases is rising and is expected to double every 20 years, reaching approximately 115.4 million by 2050. The economic consequence of this disease is immense. In 2010, it was estimated that the annual cost of dementia was $604 billion dollars (2).

Our studies have shown that sleep duration is associated with an increase in all-cause mortality (3). In this review, the relationship between sleep and cognitive function and the role sleep and sleep disorders may have in the prevention, development, and treatment of neurocognitive disorders will be considered. Underlying mechanisms are briefly examined along with the public health importance.

### Sleep Architecture

Sleep is not a quiescent state. Two processes govern sleep: the first, the homeostatic process, is often referred to as the “sleep drive,” and increases the longer the individual has been awake. The second, the circadian system, refers to the 24-h sleep-wake cycle and governs many physiological processes including production of hormones (e.g., melatonin), gene expression, and body temperature. Sleep and wake cycles are controlled by the pacemaker activity of the superchiasmic nucleus in the hypothalamus region of the brain but can be disrupted by diseases of the nervous system causing disordered sleep.

The high level of brain activity can be detected on an electroencephalogram (EEG). Sleep “architecture” consists of four different stages, and the first three stages (N1, N2, and N3) are made up of progressively deeper, non-rapid eye movement sleep (NREM). Sleep spindles along with K-complexes are defining characteristics of, and indicate the onset of, stage 2 sleep. Slow wave sleep (SWS), which may be referred to as deep sleep, occurs in Stage 3 NREM and is characterized by synchronized EEG activity. The fourth stage is characterized by rapid eye movements (REM) sleep, which can detected by a decrease in muscle activity. In general, an adult will cycle through the four stages approximately every 90–100 min during the night but the time spent in each stage changes as the night progresses.

### Sleep and Aging

Age-related changes in sleep have been well documented (4). There are changes in sleep architecture, circadian and homeostatic processes as well as changes in the susceptibility to sleep disorders. As we age, total sleep quantity and quality and sleep efficiency decline along with the amount of time spent in deep sleep. As a consequence older people often find it takes them longer to fall asleep, that they have more fragmented sleep and wake up more often and earlier (4–6). Older adults also tend to awaken less from REM sleep and more from NREM sleep than young adults and have a reduced number of sleep spindles and K-complexes in NREM [see review in Ref. (5)].

Studies indicate that aging is associated with reduced NREM and SWS (7), alongside structural brain atrophy, particularly in the frontal lobe regions (8). In a recent study, it was shown that the degree of age-related atrophy of the medial prefrontal gray matter region of the brain is significantly associated with the degree of diminution of SWS activity (9).

In a large cohort study, aging has also been shown to be associated with morning preference and a decrease in sleep efficiency. Sleep evaluations, using polysomnography, were conducted in over 2,000 subjects from the cohort who did not have sleep disorders. Detailed analysis indicated that there might be some gender differences in age-related changes in sleep: While SWS did decrease with age, the effect appeared to be more prominent in men. The study also demonstrated that in NREM, there was a decrease in the spectral power densities within slow waves and fast spindles but that for theta-alpha and beta waves, the spectral power increased with aging. In REM, aging was associated with a progressive decrease in delta waves and an increase in higher frequency waves (10).

### Sleep and Cognition (Amnestic and Non-Amnestic Function)

The term “cognition” refers to a range of mental processes, which can be grouped into two broader categories of amnestic cognitive function (ACF, referring to memory) and non-amnestic cognitive function (nACF, or non-memory). This dichotomy is important in relation to the progression from normal cognitive aging to MCI, since MCI is typically diagnosed as amnestic (aMCI) or non-amnestic (naMCI) subtype (11). Furthermore, it remains to be determined as to whether these two subtypes of MCI have different trajectories in relation to dementia (12–14).

It is well established that sleep plays a role in brain maturation and the development and maintenance of cognitive functions such as memory consolidation and learning (15). Although, the significance and functions of the various sleep stages are still not fully understood, there is, however, evidence showing that newly learned material and skills are consolidated during REM sleep (16). A link between brain cholinergic activity, timing, and density of REM sleep and cognitive functioning has also been demonstrated (17). Thus, deficiencies of REM sleep might correlate with or predict cognitive deficits in the elderly.

The formation of long-term memories requires a process of consolidation, which is facilitated by sleep. The formation of declarative (consciously recalled) memories, which are hippocampus-dependent, appears to benefit mainly from SWS (18). Recently, the focus has also been placed on stage 2 sleep and more precisely on sleep spindles, where research shows that overnight verbal memory retention is highly correlated with an increase in the number of sleep spindles (19). A hippocampal-neocortical framework of memory consolidation has been proposed whereby NREM SWS promotes the transformation of episodic memories from a state that depends on the hippocampus to one that is increasingly hippocampus-independent (20–22). Sleep deprivation after learning, however, would impair this process so that there is a greater retrieval of memories from the hippocampus (23).

### Sleep Quantity and Cognition

Sleep loss may be acute, consisting of one continuous extended wake episode, or chronic, consisting of insufficient sleep over multiple days. A substantial amount of research has been conducted to understand the former but more recently the effect of chronic sleep loss has also been investigated (24–26). In one study, self-reported short sleep, tiredness, and fatigue were more strongly associated with subjective measures of cognitive function than with objective measures (24). Findings from the Whitehall II study show that adverse changes in sleep over time (decrease from 6, 7, or 8 h, or increase from 7 or 8 h) are associated with lower scores on a variety of cognitive function tests, but not memory function (27). Subtle changes in circadian phase, for example, those that commonly occur in the general population after returning to work after later weekend sleep may also affect cognitive function. One study demonstrated that memory and verbal fluency tasks are significantly reduced on Monday morning following delayed weekend sleep (28).

### Sleep Quality and Cognition

Sleep quality is a measure of how well we actually sleep during the night. It is usually assessed via self-reported frequency of nocturnal awakenings; difficulty initiating sleep; waking up early; or waking up feeling tired, using validated tools such as the PSQI (29). Sleep quality may also play an important role in cognition. One study, in elderly women, however, found that while disturbed sleep was associated with an increased risk of developing a cognitive impairment, it was not associated with accelerated cognitive decline (30).

The Maastricht Ageing Study (MAAS) found that in middle aged and older adults subjective sleep complaints (i.e., difficulty falling asleep, waking up too early, and restless or disturbed sleep) were negatively associated with cognitive performance at the 3-year follow-up: The strongest effect being seen with early waking. The effects disappeared, however, after adjustment for depression, raising the question of whether poor quality of sleep leads directly to poor cognitive function, or whether poor sleep causes an increase in depressive symptoms, which then results in cognitive decline (31).

### Sleep and Cognitive Decline with Age

Cognitive aging appears to be a very heterogeneous process. It is associated with a decline in working memory and new episodic memory performance with relative sparing of semantic memory, recognition memory, and priming [see review in Ref. (5)]. While many neuronal changes that are associated with cognitive decline appear to begin during middle age (32), the rate of decline for individuals appears to vary considerably (33). Furthermore, while studies have demonstrated that poor sleep (quantity, quality, and efficiency) are associated with cognitive decline (34). In a recent study, looking at 3,968 male and 4,821 female white participants, aged 50 years and over, from the English Longitudinal Study of Ageing (ELSA), we demonstrated that the relationship between sleep (quantity and quality) and cognition may vary with age. Whereas in the younger age group (50–64 years), both short (<6 h per night) and long (>8 h per night) sleep were associated with lower amnestic and non-amnestic scores, in the older age group (65+ years), associations were only significant for long sleep. Furthermore, while sleep quality was associated with both amnestic and non-amnestic scores in the older age group, it was not in the younger age group. These effects were regardless of duration of sleep (35). These observations need to be fully investigated in longitudinal studies.

## Mechanisms Underlying Neurocognitive Disorders: The Role of Sleep

### Amyloid-β

Alzheimer’s disease is associated with the accumulation of amyloid-β (Aβ) in extracellular plaques in the brain. This may result from either increased production or decrease in Aβ clearance. This accumulation is thought to begin many years before the symptoms of AD are evident. It is now known that the concentrations of Aβ show diurnal variation with the levels rising during wakefulness and falling during sleep (36, 37). It has been suggested that sleep facilitates the removal of Aβ. A recent study in mice showed that during sleep, there was a 60% increase in interstitial space and a concomitant increase in the exchange of cerebrospinal fluid with interstitial fluid. This in turn increased the rate of Aβ clearance; thus facilitating the removal of these harmful waste proteins that had built up between brain cells during waking hours (38). It has been postulated that sleep disturbances may inhibit this process and may be important in the pathogenesis of AD. Indeed, evidence suggests that individuals who are cognitively normal but who have biomarker evidence of amyloid plaque formation have worse quality sleep than individuals without evidence of plague formation. The study, which used actigraphy to measure sleep, demonstrated that both sleep efficiency and wake up time after sleep onset were effected (39).

### Tau-Phosphorylation

Alzheimer’s disease is associated with the accumulation of neurofibrillary tangles (NFT) in the brain. These are composed of a highly phosphorylated form of the microtubule-associated protein tau, which unlike the non-phosphorylated form, is unable to bind to microtubules (40). Several kinases are implicated in hyper-phosphorylation of tau including c-Jun N-terminal kinase (JNK), which is also implicated in the deposition of amyloid plaque in AD brain (41).

Phosphorylation of tau via the extracellular signal-regulated kinase (ERK) pathway may occur under conditions where oxygen is lacking (hypoxia), which is a common feature of one of the most common sleep disorders known as Obstructive Sleep Apnoe (OSA). This in turn triggers neuronal degeneration and axonal dysfunction in both the cortex and brainstem (42) and may trigger cerebral amyloidogenesis (43).

### Inflammation

Inflammatory processes play a key role in the association between sleep disturbances and cardiovascular disease (44). Likewise, it is possible that inflammatory mechanisms may play a crucial role in the relationship between sleep and cognition (35) and in the sleep-associated development of neurocognitive disorders (45). Studies indicate that neuro-inflammation is characterized by the activation of microglia, which are the principal immune cells of the central nervous system (CNS) and astrocytes, which are part of the blood–brain barrier. Their function is primarily to protect the CNS, but activation of these cells leads to the release of pro-inflammatory cytokines and chemokines, which may mediate cellular damage as well (46).

In a recent study, transcriptome analysis demonstrated that in 26 individuals, 1 week of insufficient versus sufficient sleep led to a change in the gene expression of 711 genes, which were up- or down-regulated (47). Many of these genes were involved in circadian biology and sleep homeostasis as well as oxidative stress, metabolism, inflammatory, immune, and stress responses. These changes in gene expression may be involved with the negative effects of sleep loss on health.

### Melatonin Levels

Melatonin, which is produced by the pineal gland at night, plays a major role in regulation of the biological clock and the sleep–wake cycle. Studies indicate that melatonin levels are decreased in AD (48) and, reduced melatonin levels correlate with the severity of mental and sleep impairments in patient with dementia (49). Fast-release melatonin supplements given at bedtime may, however, improve cognitive and emotional performance and sleep/wake cycle maintenance in patients with MCI (50, 51).

### Genotype

#### Apolipoprotein-E Genotype

Apolipoprotein-E (APOE) genotype is the strongest genetic risk factor for AD almost half of all AD patients have at least one ϵ4 allele (52). The gene is important in lipid metabolism and cholesterol transport and polymorphic variation is associated with cardiovascular disease (53). The ϵ4 allele is also pro-inflammatory and is associated with an increase in C-Reactive Protein (CRP) in population studies (54). Individuals with this genotype accumulate more Aβ are at high risk of developing AD. Studies indicate that that effect of the adverse APOE genotype may be amplified by sleep disruption (55–59). Moreover, better sleep consolidation has been shown to attenuate the effect of *APOE* genotype on incident AD (60).

#### Circadian Clock Genes

A single night of wakefulness can alter the epigenetic and transcriptional profile of core circadian clock genes in key metabolic tissues. A recent study looked at the association between circadian locomotor output cycle kaput (CLOCK) gene rs 4580704 C/G with susceptibility of AD. It was found that among APOEϵ4 non-carriers, C carriers in CLOCK gene were associated with a high susceptibility of AD; however, among APOEϵ4 carriers the functional polymorphism of clock gene rs 4580704 C/G was not associated with AD susceptibility (61). It remains to be seen if the expression of other circadian genes is altered in dementia patients.

## Role of Sleep and Sleep Disturbances in the Development and Management of Neurocognitive Disorders

Findings from prospective studies of sleep and cognitive outcomes along with results from observational and experimental studies suggest that poor sleep is a risk factor for cognitive decline and the development of AD [see review in Ref. (62)]. Furthermore, a study in JapaneseAmerican men without dementia showed that those individuals who report Excessive Daytime Sleepiness (EDS) at baseline are twice as likely to be diagnosed with incident dementia at 3-year follow-up compared to those without EDS (63). A similar finding was also reported in elderly French men and women (64).

Sleep problems are a common occurrence in those with MCI (65) and dementia (66). The importance of sleep problems in the development, progression, management, and treatment of these disorders is still not fully recognized. The sleep issues may be underreported by the sufferer or carer at early stages but may lead to behavioral problems that are predictive of future placement of an individual in a care home (67). Nevertheless, there is growing evidence to support that sleep might be useful as a surrogate marker for Preclinical AD (68) and, there is a need to improve sleep in at risk individuals.

Individuals with dementia experience highly fragmented sleep, with periods of night-time wakefulness and frequent daytime napping (66). In general, in individuals with dementia, the proportion of sleep at night that is spent in the lighter stages of sleep is increased and there is marked decrease in time spent in the more restorative SWS. In the latter stages of dementia, emotional and behavioral changes may become more problematic and these may be associated with a decrease in observed REM sleep (69, 70). Overall, sleep in people with dementia is lighter and shorter, as well as shifted to occur earlier in the day compared to a premorbid state.

Sleep impairments and changes in sleep architecture are characteristic features of AD (71). Increased stage 1 sleep and reduced SWS, as well as decreased sleep spindles, have been reported (72, 73).

## Role of Sleep Disorders in Development of Neurocognitive Disorders

Sleep-disordered breathing (SDB) is very common in the elderly, with reported prevalence of between 24 and 42% (74). It refers to conditions, which are characterized by intermittent reduction (hypopnea) or cessation (apnea) of breathing due to narrowing of the upper airways, which in turn leads to hypoxia. Cessation of breathing during sleep causes sleep fragmentation and arousal for sleep and subsequent EDS. It is associated with an increase in neurocognitive impairments (56, 75–77).

### Sleep Apnea

The most common form of sleep apnea is OSA or obstructive sleep apnea syndrome (OSAS). OSA is characterized by recurrent sleep fragmentation and EDS, chronic intermittent hypoxia (CIH), hypercapnia, hypoventilation, peripheral and central inflammation, and changes in cerebral perfusion and glucose metabolism. Research suggests that the specific brain damage associated with OSAS could increase the risk of developing dementia (78). SDB may exacerbate cognitive dysfunction in AD (79). Indeed, positive correlations between the number of apneas occurring per hour (apnoe index) and severity of dementia in AD have been reported (80). It has been estimated that the prevalence of OSA in patients with dementing illnesses may be as high as 60% (81). But, it remains unclear, however, as to whether SDB precedes cognitive impairment or vice versa.

### Insomnia and Hypersomnia

In the population of Western Europe, the estimated prevalence of insomnia is between 10 and 35% (82). Furthermore, there is increasing evidence to suggest that insomniacs are at increased risk of cognitive decline [see review in Ref. (83)]. The detrimental performance may not be due to increased sleepiness; however, as insomniacs display a state of generalized hyperarousal with increased multiple sleep latency test (MSLT) scores (84).

Hypersomnia, which refers to either EDS or an excessive time spent sleeping, is a common feature of OSA and effected individuals have great trouble staying awake during the day. It is associated with neurocognitive impairments in both adults (85) and children (86).

### Circadian Rhythm Sleep Disorders

The term “circadian rhythm sleep disorder” describes a chronic condition in which an individual’s circadian rhythm of sleep and wakefulness is persistently or periodically out of phase with the expected environmental pattern. It is believed to occur when the biological pacemakers are decoupled from external cues as may occur for example in blind individuals. Several circadian sleep disorders have been classified including delayed sleep phase syndrome (DSPS), advanced sleep phase syndrome (ASPS), irregular sleep–wake patterns, and non-24-h sleep–wake syndrome in blind and sighted persons (87). Shift work and jet lag can also cause temporary misalignment of the circadian sleep-wake rhythm with environmental patterns.

### Circadian Rhythm Disorders

Sleep-wake disturbances are a highly prevalent and often disabling feature of AD. There are changes in several biological systems including core body temperature control, melatonin secretion, and circadian variability. It has also been suggested that sleep–wake disturbances might be due to accumulation of Aβ. It is possible that this may be a reciprocal relationship as chronic sleep deprivation increases amyloid plaque deposition, and sleep extension results in fewer plaques in experimental models (88). Furthermore, Aβ follows a diurnal pattern in that concentrations rise during wakefulness and fall during sleep (36, 37). Insomnia and circadian rhythm disorders are common causes for both caregiver burden and early institutionalization in patients for dementia (89, 90).

### REM Sleep Behavioral Disorder

Rapid eye movement sleep behavior disorder (RBD) is a sleep disturbance that commonly occurs in Dementia with Lewy bodies (DLB) (91). It is a condition in which the normal paralysis that is associated with REM sleep does not occur; sufferers are able to act out their dreams or may sleepwalk become during sleep. It usually precedes cognitive impairment by years if not decades (92).

## Presence of Sleep Disorders Could Direct the Process of Diagnosis of Neurocognitive Disorders

Poor sleep quality can be an early sign of amnestic cognitive decline (93). Furthermore, EDS may not only be an early marker but may also potentially be a reversible risk factor for cognitive decline and onset of dementia (64).

### Neuroimaging, Sleep and Neurocognitive Disorders

Amyloid plaque formation causes neuronal damage far before cognitive impairment becomes manifest (94), which makes interventions targeting these pathological changes more challenging. There is therefore a need to develop early screening techniques. Amyloid may be imaged using the PET Pittsburgh compound (PiB-Pet). A recent cross-sectional study has shown that increased brain Aβ is accompanied by lower peripheral levels of Aβ. Moreover there was a relationship between PiB binding and plasma Aβ (95). It may therefore be possible to use plasma Aβ combined with PiB binding as a risk biomarker. Although, it would be impractical to offer such screening to all individuals at risk of neurodegenerative disorders and other simpler risk markers are still needed.

Self-reported short and poor quality sleep in community-dwelling older adults is associated with greater Aβ burden (58). Likewise, in a recent study, it was shown that among older adults with MCI an increase in sleep SDB, as measured by polysomnography, was associated with greater Aβ deposition as measured by PiB-PET (59).

It is possible that sleep may normally play a vital role in removing potentially neurotoxic substances that accumulate in the awake state (38) and that sleep problems and sleep disorders may be an early marker of future neurocognitive disease development and treatment of sleep and sleep disorders might be modifier of risk in those who have a high amyloid load.

## Presence of Sleep Disorders Could Direct Management and Treatment of Neurocognitive Disorders

Research has shown that treatment of OSA via CPAP improves some aspects of cognitive function in dementia patients as well as in non-demented elderly patients with OSA (79, 96). It remains to be determined if such treatments might prevent or slow future cognitive decline, leading to dementia.

### Pharmacological Sleep Intervention in Dementia

Short-term sleep disturbances are treated using amongst others, antidepressants, benzodiazepines, and antihistamines. Benzodiazepines may decrease sleep the time taken to get to sleep “sleep latency” but drug therapies are not without side effects and long-term use may lead to increased daytime sleepiness and rebound insomnia (97).

A recent study demonstrated that MCI patients, who received fast-release melatonin at bedtime for between 15 and 60 months, exhibited significantly better performance on cognitive function tests compared to those with MCI who did not receive melatonin. Furthermore, it was reported that the group that received melatonin therapy required far less benzodiazepines to treat their associated sleep disturbances (51).

### Non-Pharmacological Sleep Interventions in Dementia

Many studies have previously focused on exercise, light or Cognitive Behavioral Therapy (CBT) for the treatment of sleep problems (45, 97). Exercise is important for physical and mental health and one study indicated that exercise may promote more restful sleep in adults (98). Bright light may be used to treat circadian rhythm disorders but there is a need to investigate this treatment further so as to develop gold standards for timing, mode of delivery, wavelength of light etc.

It remains to be seen if other interventions including CBT, to address any unhelpful beliefs and anxieties, might also be effective. Interventions may also include educating both carer and suffer with regards to how to improve and maintain good sleeping patterns. For example, by adapting the sleeping environment so that is quiet and dark, ensuring that there is a good pre-sleep wind down and ensuring that the sufferer does not spend time in the bedroom when they are not supposed to be. Social interactions might also be beneficial to sleep (97). On-going treatment for other conditions also needs to be considered as some blood pressure medication may affect sleep (99).

Further studies are required to determine if the treatment of sleep disturbances and sleep disorders may delay or prevent the development of AD and dementia. However, given the increasing population evidence to suggest that good sleep is required for general mental health and wellbeing it is important that the sufferers’ sleep does not disrupt the carers’ sleep, increasing their health burden.

### Clinical Trials of Sleep Interventions in Dementia

#### Improving Sleep Quality

A recent randomized clinical control trial demonstrated that low-intensity physical and mental activities could lead to an increase in self-reported sleep quality in older adults with self-reported cognitive and sleep difficulties (100). Further long-term studies are required with objective sleep measures to validate such studies and to see if they prevent cognitive decline.

#### Use of Prolonged-Releases Melatonin Therapy

In individuals with mild to moderate AD (*n* = 80) prolonged-release melatonin (PRM), given with standard therapy, led to a significantly better cognitive performance than those treated with placebo (101). These results contribute to the evidence that suggests that there may be a causal link between sleep and cognitive decline.

To date, it is not clear whether improvement to sleep of individuals with established sleep disturbances would delay progression of the disease. Likewise, whether treatment of early signs of sleep disturbances would lead to better outcomes and reduce the need to place the sufferer in a home. Further studies are required to determine if early interventions might (i) delay cognitive decline, (ii) improve carer stress and future health outcomes, and (iii) allow the individuals to be managed at home for longer rather than in care.

## Public Health Importance and Policy Implications

Sleep is an integral part of life. Its importance for good physical and mental health is becoming increasing apparent (6) and this may be of particular relevance for older adults. The role of sleep-related cognitive failure in major incidents and disasters has received much attention, as their consequences are often catastrophic (102, 103). As the rate of dementia is expected to increase dramatically across the world, it is important to fully understand the role that sleep and sleep disorders play in the prevention, development, and management of this disorder. It is important to increase the public awareness of the importance of sleep and to facilitate the early detection of sleep problems and disorders.

Given the emerging important relationship between sleep and cognition and cognitive impairment, assessment of sleep should be part of clinical assessment of patients presenting with cognitive impairment. To this end, the sleep study group of the Italian dementia research association (SINDem) has recently put forward recommendations for the clinical assessment and management of sleep disorders in individuals with MCI and Dementia (104). Indeed, intellectual decline associated with OSA may be misinterpreted as early dementia but may improve with appropriate OSA treatment (105). Knowledge of any underlying sleep disorders may also potentially affect the dementia diagnosis and subsequent management. Sleep apnea syndrome constitutes more than 8% of patients presenting with cognitive impairment in young adults, and it is imperative that this potentially reversible form of cognitive impairment is identified and treated early (106). This is particularly important as OSA has been identified as a risk factor for development of future dementia (107) and when treated with CPAP slower progression of the dementia was observed (108).

Studies are required to see if improvements in sleep can delay cognitive decline in at risk individuals. And research is needed to further explore the therapeutic effects but also potentially adverse effects of sleep interventions in individuals with known sleep disorders. Identifying, which individuals might be at high risk of dementia or, who may have underlying sleep problems, would allow the identification of individuals who might benefit from targeted sleep intervention. However, it is also important to appreciate that patients with dementia are at higher risk of being prescribed sleeping tablets (109–111) and, for those dementia patients with undiagnosed OSA the use of hypnotics may exacerbate cognitive impairment due to drug side effects and worsening of the sleep disorder (112).

It is necessary to explore the mechanisms underlying dementia and to determine if there may be different treatment regimens appropriate for different causative pathways. Early diagnosis of sleep disorders and sleep problems associated with dementia is required and referral routes need to be clearly agreed. There is a need for a good evidence base with regards to the effectiveness of proposed interventions in patients with dementia, which needs to be derived from large evidence–based clinical trials with objective measurements of sleep.

## Conclusion

A vast amount of research has been conducted into the effect of sleep and sleep disorders on cognition. Age-dependent changes in sleep have been well documented. Studies suggest that a “sufficient” quantity and quality of sleep are required for many aspects of both memory and non-memory cognitive function (30). The amount of sleep that constitutes “sufficient” sleep, however, continues to be debated. Nevertheless, it is generally agreed that people at the extremes of the sleep distribution, i.e., short (<5 h) and long (>9 h) sleepers are subject to cognitive deficits and accelerated cognitive aging. Proper alignment between sleep-wakefulness and internal circadian time is also crucial for optimal cognitive performance. It is accepted that many individuals with dementia have sleeping problems but despite this sleep problems in dementia and, the possibility that poor sleep may contribute to the development and progression of dementia is largely ignored. New clinical guidelines are required. Sleep disturbances and sleep disorders need to be carefully investigated using sleep history, physical examination, questionnaires etc., in both individuals with and in those at risk of development of dementia.

Further research is required to understand the associations and mechanisms involved in more detail, where the findings could have huge impacts in many areas of medicine, from normal aging to neurocognitive disorders and public health. Evidence-based research is required to develop a framework for diagnosis and treatment of sleep disorders, especially in patients with dementia.

## Conflict of Interest Statement

The author declares that the research was conducted in the absence of any commercial or financial relationships that could be construed as a potential conflict of interest.
